# Perfectionism and binge eating association: a systematic review and meta-analysis

**DOI:** 10.1186/s40337-023-00817-9

**Published:** 2023-06-26

**Authors:** María Vicent, Carolina Gonzálvez, María José Quiles, Julio Sánchez-Meca

**Affiliations:** 1 Department of Developmental Psychology and Teaching, University of Alicante, Carretera San Vicente del Raspeig s/n, 03690 San Vicente del Raspeig, Alicante, Spain; 2 Health Psychology Department, Faculty of Psychology, University Miguel Hernández of Elche, Elche, Spain; 3 Department Basic Psychology and Methodology, Faculty of Psychology, University of Murcia, Murcia, Spain

**Keywords:** Perfectionistic concerns, Perfectionistic strivings, Binge eating, Systematic review, Meta-analysis

## Abstract

**Background:**

Perfectionism is considered a vulnerability factor for eating disorders. However, the role of perfectionism in binge eating needs clarification due to notably inconsistencies between studies. The purpose to this study was to conduct a systematic review and meta-analysis to estimate the perfectionism-binge eating association.

**Method:**

Systematic review was performed according to the PRISMA 2020 statement. Four databases (Web of Science, Scopus, PsycINFO and Psicodoc) were searched to identify studies published until September 2022. The literature search yielded 30 published articles (*N* = 9392) that provided 33 independent estimations of the correlation between the two variables.

**Results:**

Random-effects meta-analysis revealed a small-to-moderate positive average effect size between general perfectionism and binge eating (*r*_+_ = .17) with a large heterogeneity. Perfectionistic Concerns showed a significant small-to-moderate relationship with binge eating (*r*_+_ = .27), whereas Perfectionistic Strivings presented a negligible relationship with binge eating (*r*_+_ = .07). Moderator analyses showed that the age, the type of the sample, the study design, and the tools for assessing both variables were statistically associated with the perfectionism-binge eating effect sizes.

**Conclusions:**

Our findings suggest that Perfectionism Concerns are closely associated with binge eating symptomatology. This relationship might be moderated by certain variables, especially by the clinical or non-clinical nature of the sample and the instrument employed to assess binge eating.

**Supplementary Information:**

The online version contains supplementary material available at 10.1186/s40337-023-00817-9.

## Abstract (plain language summary)

Perfectionism is a trait of personality comprising two facets, Perfectionistic Strivings (entails the desire to reach perfection and to pursue unrealistically high standards) and Perfectionistic Concerns (involves self-criticism, concerns over making mistakes, fears about social negative evaluation and lack of satisfaction with achievements). Perfectionist individuals have an increased risk for developing eating disorders. However, whether perfectionism or any of its facets is associated with binge eating (an episode of overeating together with a feeling of loss of control) is an unanswered question. The purpose of this systematic review and meta-analysis was to clarify this question. Our results evidenced that overall perfectionism is associated with binge eating. It means that perfectionist people are more vulnerable to developing binge eating symptomatology, although Perfectionistic Concerns entails a higher risk in comparison with Perfectionistic Strivings. Our study also provides valuable information on the aspects that might explain the variations in the results of previous studies that have analyzed the perfectionism-binge eating association.

**Supplementary Information:**

The online version contains supplementary material available at 10.1186/s40337-023-00817-9.

## Background

Binge eating is defined as “consuming abnormally large quantities of food in a discrete time period with a concurrent sense of loss of control” [2]. Considered as a transdiagnostic symptom, binge eating occurs across different eating disorders such as Binge Eating Disorder, Bulimia Nervosa, and the binge/purge subtype of Anorexia Nervosa [1]. Although central to the diagnosis of eating disorders, recurrent binge eating is also associated with both, physical and mental health problems, such as overweight or obesity, chronic pain, diabetes, hypertension, anxiety, and depression, as well as with a poor psychosocial functioning, including lower quality of life [16, 48]. Not limited to clinical population, this deviant behavior is considered a subthreshold symptom [101] with a considerable prevalence in general community, affecting 4.9% of females and 4% of males over lifetimes (see [107], for a review). Hence, binge eating represents a significant public health challenge [104], not only because of its considerable incidence, but also due to its numerous adverse consequences. Unfortunately, binge eating, as a specific symptom, remains underresearched [38].

Perfectionism, in contrast, has received a great deal of attention from research. This trait of personality is conceptualized as multidimensional, and it includes several intrapersonal and interpersonal facets [49]. There is an agreement about the existence of two higher-order perfectionism factors: Perfectionistic Strivings and Perfectionistic Concerns. This two-factor structure provides an empirical and theoretical background to compare results obtained by using different perfectionism scales and subscales [99]. Perfectionistic Strivings is considered the adaptive, or at least not maladaptive, dimension of perfectionism, and it entails the desire to reach perfection and to pursue unrealistically high standards. In contrast, Perfectionistic Concerns reflects aspects associated with self-criticism, concerns over making mistakes, fears about social negative evaluation and lack of satisfaction with achievements, representing a maladaptive component [100]. The study of perfectionism is of a huge relevance, because this trait seems to be implicated in the genesis, development, and maintenance of a wide range of psychopathologies [60]. In particular, perfectionism has long been associated to eating disorders [6]. However, while the link with Anorexia and Bulimia Nervosa is well-stablished, the association between perfectionism and Binge Eating Disorder is less clear and more research is needed to explain inconsistencies [22, 30, 57].

### Perfectionism and binge eating

Perfectionism is a core variable included across several models addressing the maintenance and persistence of binge eating behaviors (see [73], for a review). Among all these models, the Perfectionism Model of Binge Eating (PMOBE) is the only one that explain the why and how perfectionism is related with binge eating. According to this formulation, Socially Prescribed Perfectionism (a dimension described by Hewitt and Flett [49], that captures beliefs about perfectionistic demands and criticisms from significant others) promotes vulnerability to binge eating by increasing interpersonal discrepancies, low interpersonal esteem, depressive affect, and dietary restraint [85, 87]. Mackinnon et al. [65] purposed a reformulation of the model focusing on another perfectionism dimension, i.e., Concern Over Mistakes. This dimension entails the negative reactions to mistakes, interpreting them in terms of failure, and the tendency to believe that others can stop respecting us because of our mistakes [39]. Both, Socially Prescribed Perfectionism and Concern Over Mistakes are considered indicators of Perfectionistic Concerns [100]. Therefore, taken together the original and reformulated PMOBE, it seems that Perfectionistic Concerns is relevant to the occurrence of binge eating. More recently, the authors of the original PMOBE also highlighted the role of mother-daughter relationship in both perfectionism and binge eating [58]. Specifically, they evidenced that mother’s perfectionistic concerns would raise the risk of binge eating for themselves and their daughters.

Unfortunately, although there is some evidence for the PMOBE, additional direct empirical test, particularly with clinical samples, is still being necessary [73]. Moreover, the model also fails in clarifying the role of other indicators of Perfectionistic Concerns different from Socially Prescribed Perfectionism and Concern Over Mistakes, in the development of binge eating. For example, research has shown that Doubts About Actions (understood as the tendency to believe that projects are not completed to satisfaction, [39] is consistently linked with binge eating behaviors [64, 88, 89, 92]. Therefore, is the Perfectionistic Concerns second-order factor which best explains the relationship between perfectionism and binge eating, or are other specific indicators (i.e., Socially Prescribed Perfectionism, Doubts About Actions, and Concern Over Mistakes) more closely related to it?

Regarding Perfectionistic Strivings, neither the original nor the reformulated PMOBE included this dimension in the explanation of binge eating behavior. This is because results more strongly implicated Perfectionistic Concerns in comparison with Perfectionistic Strivings in binge eating. However, Mackinnon et al. [65] warned about the necessity for further research to clarify a possible role of Perfectionistic Strivings in binge eating. Certainly, the relationship between these two variables is unclear due to inconsistencies between studies’ findings. Indeed, whereas some studies have informed about a positive and statistically significant association between Perfectionistic Strivings and binge eating (e.g., [7, 11, 65, 68, 70, 85]), others reported a non-significant relationship, [5, 67, 106]. These discrepancies can be explained by different reasons, such as the dimension employed as an indicator of Perfectionistic Strivings, the sample size and type (i.e., community vs. clinical), the design of the study, etc.

Finally, it is important to notice that there are other forms of perfectionism, beyond the indicators of Perfectionistic Concerns and Perfectionistic Strivings, which might have an important explanatory role on being eating. For example, empirical studies have reported positive and significant correlations between binge eating and: Perfectionistic Self-Promotion [10, 71], a form of perfectionistic self-presentation characterized by the need to actively display one’s own perfection to others [51],self-criticism [88, 108], and parental criticisms [64] and expectations [70]. In contrast, when perfectionism is assessed as unidimensional by using a subset of items of the Bulimia subscale of the Eating Disorder Inventory (EDI, [41]) or its reviewed (EDI-2; [40]) and children versions (EDI-C; [102], the association with binge eating is unclear,some studies found a positive and significant relationship (e.g., [55, 69, 70] whereas others reported non-significant correlations (e.g., [24, 46, 80]). Hence, these noteworthy inconsistences have darkened our understanding of that relationship.

### This study

From our knowledge, no one has yet systematically studied the association between perfectionism and binge eating, as a specific symptom, using a meta-analysis. A prior meta-analysis performed by Kehayes et al. [57] with 12 longitudinal studies examined whether perfectionism would predict increased bulimic symptoms (i.e., binge eating and compensatory behaviors). Unfortunately, authors did not perform independent analysis for each bulimic symptomatology. Hence, it was not possible to draw a conclusion about the relationship between perfectionism and binge eating. As mentioned above, despite the broadly reported data of the literature supporting the association between perfectionism and eating pathology [6], the role of this personality trait in explaining binge eating behavior is not clear yet. Although previous research evidenced a positive and significant relationship between Perfectionistic Concerns and binge eating, a meta-analysis could allow a deeper understanding of this relationship by providing data about which indicator or dimension of Perfectionistic Concerns exhibit the strongest relations with binge eating. Moreover, because previous research disagrees on whether the relationship between Perfectionistic Strivings and binge eating is significant or not, a meta-analysis could help clarify this disagreement. In addition, a meta-analysis might allow to identify moderating variables that could explain those variations in results of previous studies. Last but not least, a quantitative synthesis is also needed for shedding light on the association between other forms of perfectionism and binge eating.

Therefore, the present study aimed to provide the first meta-analysis of research examining the relationship between perfectionism and binge eating. Specifically, the mean effect sizes for the relationship between binge eating and perfectionism were estimated, not only for Perfectionistic Concerns and Perfectionistic Strivings, but also for each specific indicator of these two higher-order dimensions, as well as for other forms of perfectionism. Secondly, the effects of potential moderators (i.e., the type of the sample, gender, age, nationality of the sample or measurement instruments for perfectionism and binge eating, among others), that might explain the heterogeneous outcomes were also examined.

## Method

This systematic review and meta-analysis was conducted considering the PRISMA 2020 guidelines [76].

### Eligibility criteria

Studies had to fulfil the following criteria: (a) original and quantitative investigations, (b) published in English or Spanish, (c) containing data on perfectionism and binge eating measured with validated tools, and (d) providing at least a correlation coefficient for the relationship between these two variables.

### Searching for the studies and selection process

The search was conducted on September 2022 in the databases: Web of Science, Scopus, PsycINFO and Psicodoc, using the strategy perfectionis* AND binge. Since no time limitation was established, the search period covered any document published in these databases until September 25, 2022. The search in PsycINFO and Psicodoc was performed at the same time. This is because we accessed these two databases through EBSCOhost and it was available the possibility to search on both simultaneously. We refined results to exclude documents written in any language different from English or Spanish.

**Fig. 1:**
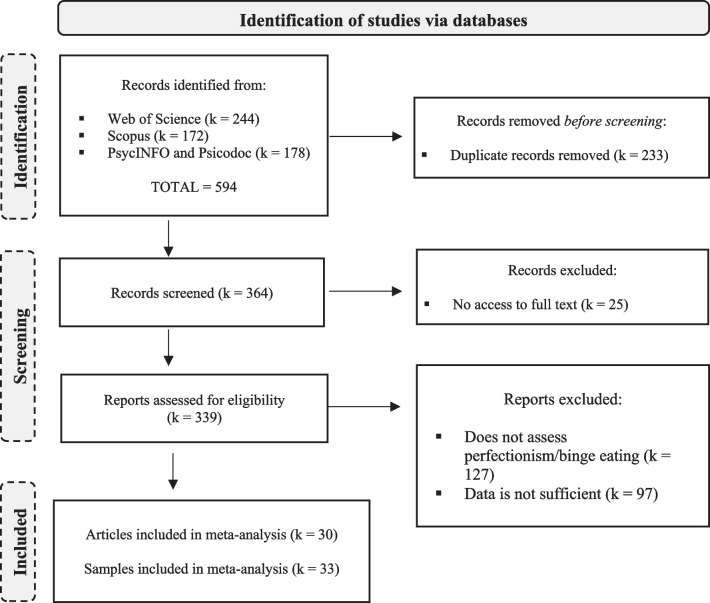
Flowchart of the study selection process in the systematic review and meta-analysis of Perfectionism and Binge Eating

Figure 1 shows a PRISMA flow diagram of the study screening and selection process. A total of 594 references were obtained from the three databases. After eliminating duplicates, we looked for the full texts of the remaining records. Of the 339 full-text documents analyzed, 127 were excluded because they did not assess perfectionism or binge eating, and 97 because data was not enough (i.e., documents did not provide a correlation coefficient). Therefore, the final sample for the meta-analysis was composed of 30 studies and 33 independent samples.

### Coding of studies

The data extracted from each study were: publication year, sample type, percentage of female participants, mean age and standard deviation of participants, country where the study was conducted, study design (longitudinal or cross-sectional), measurement tools employed to assess perfectionism and binge eating, and the correlation coefficients reported for the association between binge eating and perfectionism. In order to assess the reliability of the data extraction process, two raters independently extracted the information. Inconsistencies were resolved by consensus. Concordance between the two raters was assessed by calculating Cohen’s kappa coefficients, achieving a satisfactory average value of .96. The data that supports the findings of this study are available in the supplementary material of this article (see Additional file 1).

### Meta-analytic procedures

The effect size index in this meta-analysis was the correlation coefficient between perfectionism and binge eating measurements. From each study, all correlation coefficients reported between these two constructs were extracted. In order to normalize its distribution and stabilize the variances, the correlation coefficients were transformed into Fisher’s Z.

To avoid dependency problems, separate meta-analyses were conducted for each combination of perfectionism and binge eating measurements. In addition, correlation coefficients obtained from the same sample were averaged to conduct a global meta-analysis avoiding dependency problems. In each meta-analysis a random-effects model was assumed, as it was expected heterogeneity among the correlation coefficients. Thus, each effect size was weighted by its inverse-variance, this defined as the sum of the sampling variance and the between-studies variance. The between-studies variance was estimated by restricted maximum likelihood [13, 17]. For each meta-analysis, a forest plot was constructed and an average effect size with a 95% confidence interval with the improved method proposed by Hartung and Knapp [83]. Heterogeneity was assessed with the *Q* statistic and the *I*^2^ index. Publication bias was assessed by constructing funnel plots and by applying the Egger test and the trim-and-fill method for imputing missing effect sizes [82]. Fisher’s Zs were back-transformed to the correlation coefficient metric in order to make easy their interpretation. The influence of moderator variables was assessed by means of subgroup analyses (ANOVAs) for categorical moderators and by means of meta-regression models for the continuous ones. In all cases, the statistical significance of each moderator was tested with the improved *F-*statistic proposed by Knapp and Hartung [103]. The proportion of variance explained by each moderator was estimated with the *R*^2^ index [62]. Moderator analyses were conducted only for meta-analyses with at least 30 effect sizes. This only happened for the global correlation coefficients obtained from each of the 33 independent samples. All statistical analyses were carried out with the program Comprehensive Meta-analysis 3.3 [14].

## Results

### Description of studies

The main characteristics of the studies are presented in Appendix 1. Relevant data were obtained from 29 journal articles and one dissertation. Three studies provided data for two separate samples [5, 90, 105]. The total number of participants pooled across studies was 9392. Sample size ranged from 89 to 566. Of the 33 samples included in this meta-analysis, ten were clinical and 23 non-clinical. Most of the non-clinical samples were composed by undergraduates (*k* = 16). There were also three samples composed by adolescents or high school students, two samples of twins, one sample of emerging adults, and another of adults. Regarding the studies with clinical samples, they included patients with an eating disorder, and/or overweight or obesity. The average percentage of female participants was 82%. Twenty of the 33 samples were composed only by females, whereas three samples were exclusively composed by males. The mean age of participants through the studies was 21.6 (*SD* = 4.1; range = 11.5–41.8). The majority of the samples (*k* = 28) were conducted in North American countries (i.e., Canada and USA). Of the 30 studies, 18 used a cross-sectional design and five a longitudinal one, whereas the remaining seven studies provided both longitudinal and cross-sectional data.

**Fig. 2:**
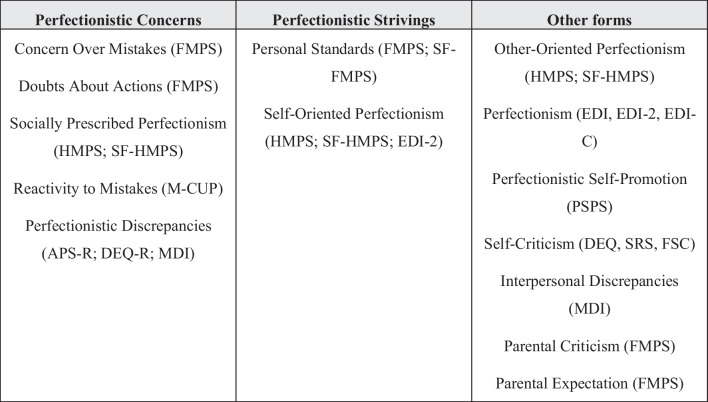
Subscales (and scales) used as indicators of Perfectionistic Concerns and Perfectionistic Strivings, as well as other forms of perfectionism

The studies used a variety of scales to assess perfectionism. Following Stoeber [99]’s classification, we considered the following subscales as indicators of Perfectionistic Concerns: Concern Over Mistakes, Doubts About Actions (Multidimensional Perfectionism Scale, FMPS, [39], Socially Prescribed Perfectionism (Multidimensional Perfectionism Scale, HMPS, [49], Multidimensional Perfectionism Scale-Short Form, SF-HMPS; [52], Reactivity to Mistakes (Measure of Constructs Underlying Perfectionism, M-CUP, [94], and Perfectionistic Discrepancies (Resconstructed Depressive Experiencies Questionnaire, DEQ-R; [3], Almost Perfect Scale-Revised, APS-R, [91], Multidimensional Discrepancy Inventory, MDI; Flett and Hewitt [31]). On the other hand, Personal Standards (FMPS; [39], SF-FMPS; [21] and Self-Oriented Perfectionism (HMPS [49], SF-HMPS; [52] subscales were considered as indicators of Perfectionistic Strivings. Some studies tended to combine more than one measure of perfectionism to assess these two-higher order dimensions. Other forms of perfectionism, such as Other-Oriented Perfectionism (HMPS [49], SF-HMPS; [52], Parental Criticisms, Parental Expectations (FMPS, [39], Perfectionistic Self-Promotion (Perfectionistic Self-Presentation Scale, PSPS, [51], Self-criticism (Depressive Experiencies Questionnaire, DEQ, [8],Self-Rating Scale, SRS; [54],Self-Criticism/Self-Reassurance Scale, FSC; [42], and the Perfectionism subscale from the EDI [41] and its different versions (EDI-2, [40], EDI-C; [102] were excluded from this classification. It is important to notice that, although the Perfectionism subscale of the EDI is a unidimensional measure of perfectionism, a two-dimensional structure representing Self-Oriented Perfectionism and Socially Prescribed Perfectionism was identified [86]. Consequently, when studies considered a two-dimensional structure (this was the case of Lampard et al. [59], and [85], each subscale, Self-Oriented Perfectionism and Socially Prescribed Perfectionism, was considered, respectively, as an indicator of Perfectionistic Strivings and Perfectionistic Concerns. Figure 2 shows a classification of the measures employed by the studies included in the meta-analysis as indicators of Perfectionistic Concerns, Perfectionistic Strivings, and other forms of perfectionism.

Binge eating symptoms were assessed through different tools. Two studies assessed the frequency of binge eating behavior by using the Eating Disorders Examination (EDE; [27]), an interview widely used to assess eating disorder symptoms. In addition, in Jones and Crowther’s [55] study binge eating was assessed by asking the participants whether they engaged or not in binging during the previous year. Apart from these three investigations, studies included in this meta-analysis used self-report measures to assess binge eating tendencies. The EDE-Q [25], which is the self-report form of the EDE, and recent versions of that questionnaire, such as the EDE-Q 6.0 [26] and the version for children (ChEDE-Q, [53]), were the most used measures to assess binge eating (*k* = 9). Another measure widely employed was the Bulimia subscale of the EDI [41] and EDI-2 [40] (*k* = 7). As that subscale assesses symptoms of bulimia, purging and binge eating, authors employed different subsets of the items focused on binge eating. Other examples of self-report scales employed were a 7-item version of the Binge Eating subscale from the Eating Disorder Diagnostic Scale (EDDS; [97] (*k* = 3), and the Binge Eating Scale (BES; [45]) (*k* = 2), between others.

### Average effect size and heterogeneity

**Fig. 3:**
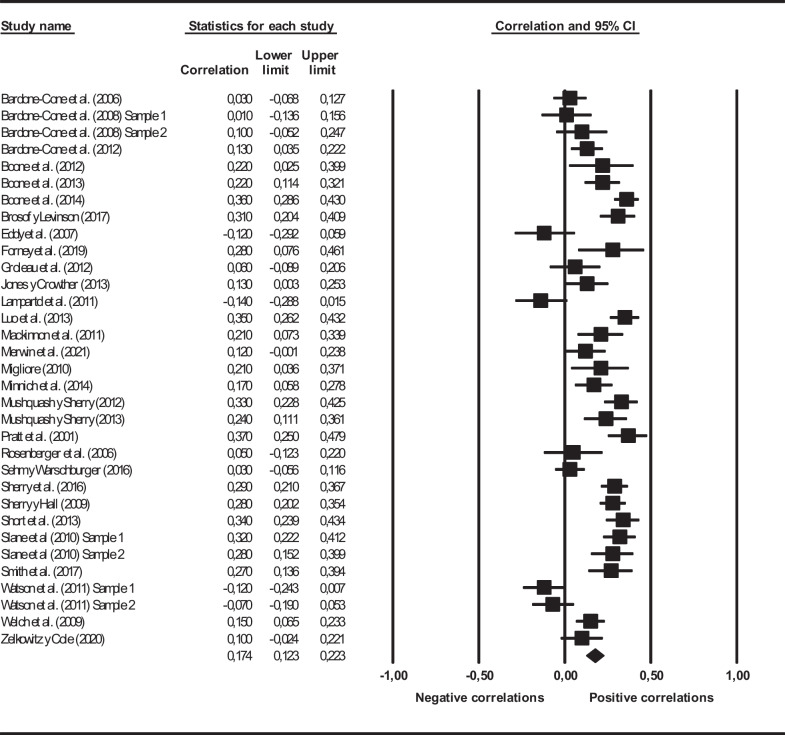
Forest plot of the association between Perfectionism and Binge Eating for the global correlation coefficients obtained from the 33 independent samples

The association between perfectionism and binge eating was estimated in this meta-analysis. Figure 3 presents a forest plot of the correlations found between both variables in each individual study and their 95% CI for the 33 independent samples. The forest plot also presents the average correlation coefficient resulting from pooling all the studies, reaching a value of *r*_+_ = .17 (95% CI = .12–.22, *k* = 33). To help to interpret the correlation coefficient magnitude, we followed Cohen’s [19] guidelines for small, medium, and large effects (*r* = .10, .30, and .50, respectively). Thus, the average global correlation coefficient between perfectionism and binge eating exhibited a positive, small-to-moderate magnitude. The individual effect sizes exhibited a large heterogeneity, *Q*(32) = 196.54, *p* < .001, *I*^2^ = 83.7% (Table 1).

**Table 1:** Summary of overall effect sizes for the relationship between perfectionism dimensions and binge eating

Variable	*k*	*N*	*r*_+_	95% CI	*Z*	*p*	*Q*_*T*_	*I*^*2*^	*p*	*Tau*^*2*^
Perfectionistic concerns^a^	22	6593	.27	.23–.31	12.74	< .001	61.57	65.89	< .001	.007
Concern over mistakes^b^	10	2629	.28	.22–.33	9.86	< .001	17.93	49.81	.04	.004
Socially prescribed perfectionism	11	3705	.23	.17–.28	8.70	< .001	24.36	58.92	.007	.004
Doubts about actions	4	1448	.34	.28–.39	11.04	< .001	4.27	29.66	.23	.001
Perfectionistic discrepancies	1	–	–	–	–	–	–	–	–	–
Perfectionistic strivings^c^	13	3525	.07	.01–.13	2.18	.03	41.02	70.75	< .001	.009
Personal standards	5	966	.11	− .003–.22	1.91	.06	11.99	66.64	.02	.01
Self-oriented perfectionism	7	2380	.09	.02–.15	2.62	.009	10.33	51.61	.07	.003
*Other forms of perfectionism*										
Perfectionism	9	2727	.09	.03–.16	2.87	.004	21.57	62.90	.006	.006
Self-criticism	2	–	–	–	–	–	–	–	–	–
Other-oriented perfectionism	2	–	–	–	–	–	–	–	–	–
Interpersonal discrepancies	1	–	–	–	–	–	–	–	–	–
Parental criticism	1	–	–	–	–	–	–	–	–	–
Parental expectation	1	–	–	–	–	–	–	–	–	–
Perfectionistic self-promotion	1	–	–	–	–	–	–	–	–	–
Perfectionistic self-presentation	1	–	–	–	–	–	–	–	–	–
Total	33	9392	.17	.12–.22	6.69	< .001	196.54	83.72	< .001	.02

*k* values for Perfectionistic Concerns and Perfectionistic Strivings also include those studies that combined more than one measure of these higher order dimensions

*k*, number of studies; *N*, total number of participants in the *k* samples; *r*_**+**_, weighted mean correlation coefficient; CI, confidence interval; *Q*_T_, heterogeneity statistic of the effect sizes; *I*^*2*^, percentage of heterogeneity; *Tau*^2^, between-studies variance

^a^Perfectionistic Concerns assessed as aggregate of Concern Over Mistakes, Reactivity to Mistakes, Socially Prescribed Perfectionism, Doubts About Actions and Perfectionistic Discrepancies

^b^Subscale Reactivity to Mistakes of the M-CUP (*k* = 1) was also considered as Concern Over Mistakes, due to the theoretical similarity between the two subscales

^c^Perfectionistic Strivings assessed as aggregate of Personal Standards and Self-Oriented Perfectionism

Separate meta-analyses were also conducted to examine the associations between perfectionism and binge eating but establishing differences between the two higher-order factors of perfectionism (i.e., Perfectionistic Concerns and Perfectionistic Strivings) as well as for each specific perfectionism dimension. Because the data about the relationship between some perfectionism dimensions and binge eating were underrepresented, a minimum of studies (*k* ≥ 3 independent studies) was stablished to calculate effect sizes for each specific dimension. However, those data were considered for the analyses of their respective higher-order dimension, i.e., Perfectionistic Concerns or Perfectionistic Strivings. Taking into account this criterion, in the case of other forms of perfectionism, effect sizes were analysed only for the perfectionism subscale of EDI. However, all measures were considered to calculate the total effect about the relationship between perfectionism and binge eating. Table 1 presents the average effect sizes for each meta-analysis together with their 95% confidence intervals and heterogeneity statistics. The global dimension of Perfectionistic Concerns and its respective indicators (Concern Over Mistakes, Socially Prescribed Perfectionism) exhibited a small-to-moderate positive relationship with binge eating (*r*_+_ = .27, .28, and .23, respectively), with the exception of Doubts About Actions that exhibited a moderate positive relationship (*r*_+_ = .34). All of these average correlation coefficients were statistically significant (*p* < .001). The global dimension of Perfectionistic Strivings and its indicator ‘Self-Oriented Perfectionism’ presented an average effect size under de cut-off value of .10 (*r*_+_ = .07 and .09, respectively), leading to a negligible relationship with binge eating. In addition, the indicator ‘Personal Standards’, although achieved a small positive relationship with binge eating, it did not reach statistical significance (*r*_+_ = .11; 95% CI: − .003–.22). Regarding the association between perfectionism assessed by using the EDI and binge eating, it did not reach a correlation > .10 according to Cohen’s criteria (*r*_+_ = .09). The heterogeneity test (*Q*) was statistically significant for all meta-analyses, except for the indicator ‘Doubts About Actions’ (*p* = .23). The heterogeneity indices (*I*^2^) ranged from 49.8 to 70.8%, suggesting the existence of moderate-to-large heterogeneity among the individual effect sizes.

### Moderator analyses of the relationship between perfectionism and binge eating

Moderator analyses were conducted for the 33 independent global correlation coefficients obtained from each study. Moderator analyses were not carried out for the other more specific meta-analyses because none of them had 30 effect sizes at least. Categorical moderators analyzed by means of subgroup analysis (ANOVA) were the average age of the sample (adolescents 11–17, young adults 18–25, and adults > 25), sample type (clinical, non-clinical undergraduates, and non-clinical others), sample size (*N* = 80–200, 201–400, > 400), *country* (Australia, Belgium, Canada, USA, and Canada + UK), study design (cross-sectional vs. longitudinal), and instruments (perfectionisms scales, and binge eating scales). Continuous moderators analyzed by means of simple meta-regressions were the percentage of females, mean age of the sample and the publication year.

Table 2 presents the results of the ANOVAs conducted on the categorial variables. Five moderator variables showed a statistically significant association with the effect sizes: (a) the age, (b) the type of the sample used in the study, (c) the design, (d) the measurement instrument used to assess perfectionism, and (c) the measurement instrument used to assess binge eating. The age of the samples, categorized into adolescents, young adults, and adults, exhibited a statistically significant relationship with the effect sizes (*p* = .02; *R*^2^ = .20). Studies with samples composed by young adults showed a moderate-to-small association between perfectionism and binge eating (***r***_**+**_ = .22), in comparison with adolescents (*r*_+_ = .14) and adults (*r*_+_ = .03). In addition, stronger associations were observed when non-clinical samples (*p* = .001; *R*^2^ = .56), either undergraduates (***r***_**+**_ = .22) or others (***r***_**+**_ = .24), were employed, compared to clinical samples (*r*_**+**_ = .04). Regarding the study design (*p* = .004; *R*^2^ = .30), studies with longitudinal data did not reach a statistically significant perfectionism-binge eating association (*r*_+_ = .03), whereas cross-sectional studies reported a positive, significant relationship (***r***_**+**_ = .21). In the same line, the perfectionism scale exhibited a statistically significant association with the effect sizes (*p* = .03; *R*^2^ = .32). Specifically, the perfectionism scales than showed a positive, statistically significant average correlation with binge eating were FMPS versions (*r*_+_ = .26), HMPS versions (*r*_+_ = .21), and several measures combined (*r*_+_ = .15). Finally, the employed tool for assessing binge eating also exhibited a statistically significant association with the effect sizes (*p* < .001; *R*^2^ = .90). Moderate-to-small average correlations between perfectionism and binge eating were obtained for EDI versions (*r*_+_ = .30), frequency of binge eating (*r*_+_ = .30), MEBS (*r*_+_ = .30), measures combined (*r*_+_ = .28), EPSI (*r*_+_  = .28), BES (*r*_+_ = .26), EDDS (*r*_+_ = .23), and Binge Scale (*r*_+_ = .15).

**Table 2:** Results of the weighted ANOVAs for the influence of categorical variables on the effect sizes

Moderator variable	*k*	*r*_+_	95% CI		ANOVA results
			*r*l	*r*u	
*Age*					*F*_(2,28)_ = 4.48, *p* = .02 *R*^2^ = .20 *Q*_E(28)_ = 160.40, *p* < .001
Adolescents (11–17 years)	4	.14	.004	.28	
Young adults (18–25 years)	20	.22	.15	.27	
Adults (> 25 years)	7	.03	− .08	.14	
*Sample type*					*F*_(2,28)_ = 14.90, *p* = .001 *R*^2^ = .56 *Q*_E(28)_ = 103.84, *p* < .001
Clinical	10	.04	− .05	.12	
Non-clinical undergraduates	16	.22	.16	.28	
Non-clinical others	7	.24	.15	.33	
*Design*					*F*_(1, 29)_ = 9.94, *p* = .004 *R*^2^ = .30 *Q*_E(29)_ = 132.19, *p* < .001
Cross-sectional	26	.21	.17	.26	
Longitudinal	7	.03	− .07	.12	
*Perfectionism scale*					*F*_(5, 25)_ = 3.12, *p* = .03 *R*^2^ = .32 Q_E(25)_ = 109.25, *p* < .001
EDI versions^a^	8	.03	− .06	.12	
FMPS versions^b^	11	.26	.19	.33	
HMPS versions^c^	5	.21	.10	.32	
M-CUP	1	.28	− .02	.53	
PSPS	1	.22	− .02	.42	
Measures combined^d^	7	.15	.06	.24	
*Sample size*					*F*_(2, 28)_ = 1.56, *p* = .23 *R*^2^ = .04 *Q*_E(28)_ = 171.72, *p* < .001
80–200	10	.09	− .004	.19	
201–400	13	.19	.11	.26	
> 400	8	.20	.11	.30	
*Geographic location*					*F*_(5, 25)_ = 1.56, *p* = .21 *R*^2^ = .10 *Q*_E(25)_ = 136.35, *p* < .001
USA	15	.16	.09	.23	
Canada	10	.17	.08	.25	
Belgium	3	.28	.12	.42	
Australia	1	− .14	− .41	.16	
Canada and UK	1	.29	.03	.51	
*Binge eating scale*					*F*_(11, 19)_ = 8.99, *p* < .001 *R*^2^ = .90 *Q*_E(29)_ = 28.84, *p* = .07
EDE versions^e^	11	.02	− .03	.07	
EDI versions^f^	7	.30	.24	.35	
EDDS	3	.23	.14	.32	
BES	2	.26	.15	.37	
MEBS	2	.30	.19	.40	
Measures combined^g^	2	.28	.17	.38	
Binge Scale	1	.15	.008	.29	
Binge = 1/No Binge = 0	1	.13	− .04	.29	
BMS	1	− .12	− .32	.09	
EPSI	1	.28	.05	.48	
ESS	1	.13	− .02	.27	
Frequency of binge eating	1	.30	.21	.47	

*K*, number of studies; *r*_+_, mean effect size; *rl* and *ru*, 95% lower and upper confidence limits around *r*_+_; *R*^2^, proportion of variance accounted for by the moderator variable; *F*, statistic to test the statistical significance of the moderator proposed by Knapp and Hartung; *Q*_E_, Statistic for testing the model misspecification; M-CUP, Measure of Constructs Underlying Perfectionism; PSPS, Perfectionistic Self-Presentation Scale; EDDS, Eating Disorder Diagnostic Scale; BES, Binge Eating Scale; MEBS, Minnesota Eating Behavior Survey; BMS, Bulimic Modeling Scale; EPSI, Eating Pathology Symptom Inventory; ESS, Eating Self-Efficacy Scale

^a^Studies that use the Eating Disorder Inventory or the Eating Disorder Inventory-2 to assess perfectionism

^b^Studies that use the Frost Multidimensional Perfectionism Scale or its brief version

^c^Studies that use the Hewitt Multidimensional Perfectionism Scale or its brief version

^d^Studies where perfectionism score is calculated by combining different measures

^e^Studies that use any of the following tools: the Eating Disorder Examination Questionnaire, the Eating Disorders Examination, the Child Eating Disorder Examination-Questionnaire, or the Eating Disorder Examination Questionnaire 6.0

^f^Studies that use the Eating Disorder Inventory or the Eating Disorder Inventory-2 to assess binge eating

^g^Studies where binge eating score is calculated by combining different measures

Simple meta-regressions conducted on continuous moderators such as the percentage of females, mean age and publication year are presented in Table 3. None of these moderators exhibited a statistically significant association with the effect sizes.

**Table 3:** Results of the simple weighted meta-regressions of continuous moderators on the effect sizes

Moderator	*k*	*b*_*j*_	*F*	*p*	*Q*_*E*_	*p*	*R*^*2*^
Female %	31	− .0005	.37	.55	187.67	< .001	0
Mean age	30	− .003	.40	.53	181.51	< .001	0
Year	31	.012	2.98	.10	178.38	< .001	.06

*K*, number of studies; *bj*, unstandardized regression coefficient; *F*, statistic to test the statistical significance of the moderator; *Q*_*E*_, *Q* statistic for testing the model misspecification; *R*^*2*^, proportion of variance accounted for by the moderator variable

### Analysis of publication bias

**Fig. 4:**
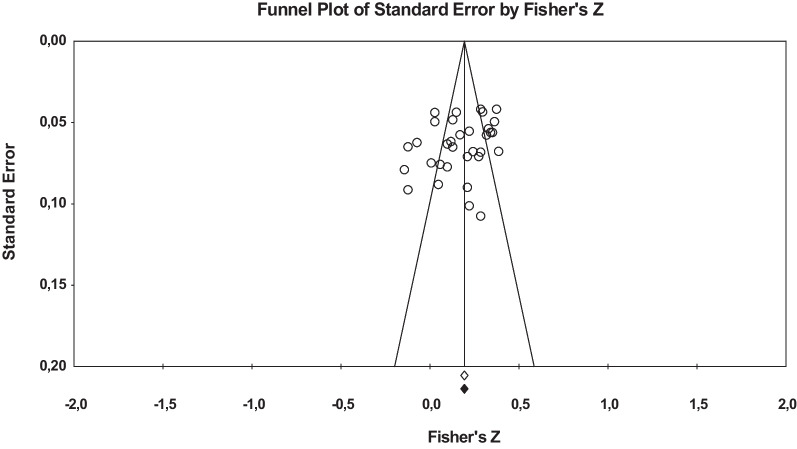
Funnel plot of the association between Perfectionism and Binge Eating for the 33 global correlation coefficients (transformed into Fisher’s Z). Empty diamond represents the average Fisher’s Z for the 33 original effect sizes. Black diamond represents the average Fisher’s Z for the original effect sizes plus the imputed ones with the trim-and-fill method. As this method did not impute any additional effect size, the empty and black diamonds coincide

All the studies included in this meta-analysis were published papers (with one exception only), so statistical analyses were carried out to determine whether publication bias might be a threat to the validity of the results of the meta-analysis. Figure 4 presents a funnel plot relating the effect sizes with their standard errors. The Duval and Tweedie trim-and-fill method did not impute missing studies to symmetrize the funnel plot. In addition, the Egger’s test [95] applied to the intercept of a simple regression model of the effect sizes reached a non-statistically significant result [*t*(31) = 1.72, *p* = .09]. Therefore, publication bias can be discarded as a threat to the meta-analytic results.

## Discussion

The present meta-analysis of 30 studies, 33 samples, and 9392 participants represents the most comprehensive test of the perfectionism-binge eating relationship to date. The study found a positive and statistically significant correlation between overall perfectionism and binge eating, indicating that higher levels of perfectionism were associated with higher levels of binge eating. Specifically, a correlation coefficient of *r*_+_ = .17 was obtained, which, according to Cohen’s [19] guidelines, can be interpreted as reflecting a relationship of small magnitude. This positive and significant link was consistent across the two higher-order dimensions—Perfectionistic Concerns and Perfectionistic Strivings–, as well as its respective indicators (except for Personal Standards) and the EDI-Perfectionism subscale. However, variations regarding effect sizes associated to the correlation coefficients ranging from .07 to .34 were observed. Specific relationships are now discussed in detail.

### Perfectionistic concerns and binge eating

Perfectionistic Concerns has been traditionally considered a clearly maladaptive component of perfectionism [100]. Thus, as expected, Perfectionistic Concerns showed a positive and significant association, of a moderate-to-small magnitude (*r*_+_ = .27), with binge eating. This link was consistent across the three indicators of Perfectionistic Concerns evaluated in this study—Concern Over Mistakes (*r*_+_ = .28), Socially Prescribed Perfectionism (*r*_+_ = .23), and Doubts About Actions (*r*_+_ = .34)—with small-to-moderate positive correlations. These findings provide additional empirical support for the original [85, 87] and reformulated [65] PMOBE according to which Socially Prescribed Perfectionism and Concern Over Mistakes promote vulnerability to binge eating by generating four binge eating maintenance variables (i.e., interpersonal discrepancies, low interpersonal esteem, depressive affect, and dietary restraint) that are viewed as both precursors and sequels of binge eating. However, neither the original nor the reformulated PMOBE considered Doubts About Actions as a possible explanatory factor of binge eating. In this sense, considering that Doubts About Actions-binge eating link was the only one that reached a moderate effect size, our results advise expanding the PMOBE including the possible role of Doubts About Actions, which could increase our understanding of binge eating beyond Socially Prescribed Perfectionism and Concern Over Mistakes. Doubts About Actions is defined as a nagging sense of uncertainty regarding the quality of one’s performance [39]. These annoying self-doubts might also confer vulnerability to binge eating through the four triggers stablished by the PMOBE. People high in Doubts About Actions appears to be more likely to experience interpersonal problems such as need for social approval, lack of sociability, interpersonal sensitivity, social anxiety, etc. [20, 23, 81]. That social disfunction vulnerability might predispose individuals with high levels of Doubts About Actions to perceive more interpersonal discrepancies and poor interpersonal esteem. Additionally, the frequent perception of being disappointing others might explain why doubting one’s performance is closely related with depressive symptoms [93], resulting, in turn, in self-defeating behaviors, such as binge eating, as a coping response.

On the other hand, both, the need for social approval and dietary restraint are implicated factors in the pathogenesis of binge eating [9, 56]. Under the influence of mass media, peers, and family, individuals high in Doubts About Actions, due to their frequent self-evaluation, may feel particularly pressured to be thin and engage in upward social comparison. That pursuit of the thin ideal, with subsequent body dissatisfaction, dieting restrictions, and unhealthy control eating behaviors predispose the onset of binge eating disorders [98]. That is, individuals characterized by high levels of Doubts About Actions may also unsuccessfully attempt to restrain their eating to gain social approval from significant others (e.g., family, peers, coaches, teachers…), compensating then that caloric deprivation by binge eating. Interestingly, Kim et al. [58] found that mothers and daughters’ Perfectionistic Concerns (conceptualized as the sum of Doubts About Actions and Concern Over Mistakes) play an important role in binge eating. Of the two indicators of mothers’ Perfectionistic Concerns, Doubts About Actions was the only one significantly correlated with daughter’s binge eating. In this sense, it may be that binge eating arises in daughters as a consequence of their attempts to diet to alter their shape to please their hypercritical mothers.

### Perfectionistic strivings and binge eating

Contrary to Perfectionistic Concerns, the maladaptive or adaptive nature of Perfectionistic Strivings has been a matter of discussion (e.g., [33, 61, 75]. In this sense, our results evidenced that this higher-order dimension would be positively and significantly associated with binge eating (*r*_+_ = .07), although in a lesser magnitude than Perfectionistic Concerns. Of the two Perfectionistic Strivings indicators assessed in this study, only the Self-Oriented Perfectionism-binge eating link reached the statistical significance. However, it is worth note that the negligible average effect size obtained for the Self-Oriented Perfectionism-binge eating correlation (below .10) indicates that this association, although statistically significant, has no practical relevance. That statistical significance reached by both, Perfectionistic Strivings and Self-Oriented Perfectionism, might be explained by the large sample size; *N* = 3525 and 2380 participants, respectively, in comparison with the *N* = 966 participants for the Personal Standards calculations. Overall, these results suggest that the tendency to set extremely high standards and motivation to attain perfection has no relevant effect on this maladaptive eating behavior.

### Other forms of perfectionism and binge eating

Other forms of perfectionism not classified as indicators of Perfectionistic Concerns and Perfectionistic Strivings were also considered in this meta-analysis. Unfortunately, the lack of studies prevented the analysis of all those other forms of perfectionism except for the perfectionism subscale of EDI. Surprisingly, EDI-Perfectionism relationship with binge eating reached a significant association but with values below .10. Again, it means that this relationship, although statistically significant, seems to be negligible for practical purposes. This is an interesting result, since the EDI-Perfectionism subscale was designed to measure perfectionism associated with eating disturbances. In this sense, Chang et al. [18] compared three measures of perfectionism (FMPS, HMPS and EDI-perfectionism) to identify the strongest unique perfectionism predictors of eating disturbances and health behaviors. Surprisingly, EDI-Perfectionism did not emerge as a significant unique predictor of any form of eating disturbances. Therefore, according to the authors, it calls into question studies that have employed that subscale as the only measure of perfectionism because their findings may have been subjected to the confusion of perfectionism (as a symptom of eating disturbances) with disturbed eating outcomes. Further, although the EDI-Perfectionism has been widely employed to assess perfectionism as a unidimensional construct, some research has evidenced that it is best represented by a multidimensional structure with items capturing a partial representation of Self-Oriented Perfectionism and Socially Prescribed Perfectionism (e.g., [86]. In this sense, we agree with Hewitt's et al. [50] idea about the multidimensionality of perfectionism and therefore it must be assessed using a multidimensional measure.

### Moderator variables

Even though no evidence of publication bias was found as a threat to the validity of our results, a large heterogeneity among the individual effect sizes was reported. Five of the ten moderator variables analyzed in the meta-analysis had a statistical influence on the effect sizes for the perfectionism-binge eating correlations: the age, the type of the sample, the design, and the measurement instrument used to assess perfectionism and binge eating. Regarding the age of the sample, our results obtained strongest associations in young adult (18–25 years) samples than in adolescents and adults. This result makes sense considering that binge eating seems to be more common in young adulthood [107]. According to that, preventive interventions should be addressed particularly to adolescents and young adults.

Secondly, perfectionism was more strongly associated with binge eating when non-clinical samples were used in comparison with clinical samples. A possible explanation of this result would be that the association between perfectionism and binge eating was a consequence of an underlying third variable; fasting. It is important to highlight that clinical samples in this meta-analysis were comprised of individuals with Bulimia or other clinical conditions (Binge Eating Disorder, overweight or obesity) associated to binge eating symptomatology. Binge eating does not distinguish eating disorders, as it is a common symptom even in a subtype of Anorexia Nervosa [1]. However, under-eating and starvation predominate in anorectic patients in comparison with other clinical conditions—indeed, resulting in extremely low body weight. Thus, the perfectionism-binge eating link would be attributable to the presence of fasting among those who binge [34]. This fact also might explain why perfectionism has been considered as a specific risk factor for Bulimia and Anorexia Nervosa, but not for Binge Eating Disorder [28, 29], and why women with Anorexia Nervosa tend to be characterized by higher levels of perfectionism when compared with patients with Bulimia Nervosa [6, 37]. However, this result must be interpreted very cautiously as the negligible perfectionism-binge eating relationship found in clinical samples could be explained by the lack of variability in the scores reported by participants. Furthermore, an alternative explanation is insufficient statistical power, given that the sample size of studies with clinical samples is smaller in comparison with those with community samples. In the same line, considering that four of the seven samples with longitudinal data were composed by clinical population, it may also explain the weakest association between perfectionism and binge eating in studies with a longitudinal design than with a cross-sectional one.

Furthermore, results showed that the EDI-Perfectionism subscale was the least sensitive tool in identifying relationships between perfectionism and binge eating symptomatology. As mentioned above (see “Other forms of perfectionism and binge eating” Section), the EDI-Perfectionism consists of six items originally developed to assess perfectionism in the context of eating disturbances. Even though the subscale was created, and has been mostly employed, as a unidimensional measure of perfectionism, several studies advert that half of the items appear to reflect Self-Oriented Perfectionism whereas the other reflect Socially Prescribed Perfectionism (e.g., [86]. This tendency to jointly assess well-differentiated facets of perfectionism in a brief measure that partially represents more complex constructs could explain this unexpected result.

Finally, findings revealed that the perfectionism-binge eating association varied considerably depending on the tool employed to assess binge eating, being, in fact, the moderator variable that explained the highest proportion of variance (*R*^2^ = .90). Thus, even though most of the binge eating assessment tools provided small-to-moderate correlation coefficients, studies using EDE/EDE-Q versions reported non-significant correlations, and the study of Eddy et al. [24], using the BMS [96], reported a negative perfectionism-binge eating correlation. Unfortunately, the small number of studies per subgroup advise caution in interpreting these effect sizes. Therefore, to avoid biased or inaccuracy conclusions, we are going to focus on discussing mean effect sizes obtained from more than 3 studies (i.e., EDE/EDE-Q versions and EDI versions). Eleven studies analyzed the relationship between perfectionism and binge eating using different versions of the EDE or the EDE-Q. The EDE is an expert semi-structured interview that provides frequency data on key features of eating disorders. Although the EDE allows to assess both objective and subjective overeating, researchers usually resort to this interview to analyze objective binge eating operationalized as the number of episodes in the past 28 days [46, 59]. This tool is considered the gold standard for the diagnosis of Binge Eating Disorder [72]. Unfortunately, it has certain disadvantages for research purposes. For instance, it requires extensive interviewer training and familiarity with the tool to ensure competency as well as valid and reliable results [77]. These shortcomings were addressed by the EDE-Q, a self-report measure based on the EDE that assesses symptoms of eating disturbances over the past 28 days, providing investigators with a practical and inexpensive evaluation method. Using the EDE-Q it is possible to assess objective binge eating with two items that ask participants “how many times have you eaten what people would regard as an unusually large amount of food given the circumstances?” and “did you have a sense of having lost control over your eating at the time you were eating?”. However, these manner of analyzing objective binge eating through the EDE-Q has led to some controversy, suggesting that subjects, particularly those diagnosed with a Binge Eating Disorder, should be instructed in the construct of binge to avoid ambiguities in their interpretation [44].

The EDI-Bulimia is a 7-item self-report measure to assess the tendency toward episodes of uncontrollable overeating and the impulse to engage in self-induced vomiting. One problem of using this subscale for assessing binge eating is that, whereas there is an item clearly reflecting purge tendencies (“I have the thought of trying to vomit in other to lose weight”) and four items clearly addressing binge tendencies (“I stuff myself with food”, “I have gone on eating binges where I have felt that I could not stop”, “I think about bingeing”, and “I eat moderately in front of others and stuff myself when they are gone”), the remaining two items do not necessary reflect binge or purge tendencies (“I eat when I am upset” and “I eat or drink in secrecy”). Thus, this has led to disagreements between investigations when selecting the number of items (four to six) to assess binge eating.

One important question about our findings is why EDE/EDE-Q versions where the least sensitive instruments in identifying perfectionism-binge eating association (*r*_+_ = .02), whereas the EDI-Bulimia was one of the most sensitive tools to capture this relationship (*r*_+_ = .30). A tentative explanation may be that eight of the eleven studies using the EDE or EDE-Q versions were developed with clinical samples, whereas all studies using the EDI-Bulimia were based on community samples. Therefore, the lack of dispersion of data might justify weak correlation coefficients between perfectionism and binge eating. An alternative explanation involves the fact that EDE/EDE-Q provide a clear differentiation between objective and subjective overeating, whereas EDI-Bulimia does not distinguish both constructs. Thus, whereas some items of EDI-Bulimia are referred to objective binge episodes (i.e., “I have gone on eating binges where I have felt that I could not stop”), others are assessing feelings or thoughts that might emerge even in the absence of consuming a large amount of food (i.e., “I think about bingeing”).

### Limitations and suggestions for future research

It is important to highlight that this study has several limitations. First, it is possible that this meta-analysis has captured more comprehensively Perfectionistic Concerns (comprised of four indicators: Concern Over Mistakes, Socially Prescribed Perfectionism, Doubts About Actions, and Perfectionistic Discrepancies) than Perfectionistic Strivings (compromised by only two indicators: Personal Standards and Self-Oriented Perfectionism). Secondly, most research on the perfectionism-binge eating link is on trait perfectionism. Thus, future studies should address this gap analyzing possible implications of other forms of perfectionism, such as perfectionistic self-presentation [51] or automatic thoughts [32] on binge eating symptomatology. Moreover, most of the studies included were performed in USA or Canada, which might limit the generalization of our results. Because perfectionism’ outcomes might be influenced by sociocultural factors [66], an important avenue for future research would be to analyze how perfectionism is related with binge eating across cultures and ethnic minorities. Similarly, more longitudinal studies, not only with clinical participants but also with community samples are also required to test if perfectionism confers risk for binge eating over time. Also, future investigators should consider adopting a multidimensional approach as a more comprehensive assessment of perfectionism rather than using unidimensional measures such as the EDI-Perfectionism. Finally, considering the huge amount of variance explained by the binge eating tool employed, researchers must be particularly thoughtful in selecting the measurement instrument. In this sense, although the distinction between subjective and objective binges seems not to be clinically useful [74], we suggest that future research investigates if the perfectionism-binge eating link would be the same regardless of subjective or objective overeating is considered (Additional file 1).

## Conclusions

This meta-analysis sheds light on the perfectionism-binge eating link, extending current knowledge about the association of this trait of personality and psychopathology [60]. Decades of research suggest that perfectionism is a central core of eating disturbances, and our study corroborates that binge eating is not an exception [22, 30, 57]. In fact, all the perfectionism indicators analyzed in this study were positively and statistically associated with binge eating, except for Personal Standards whose association did not reach the statistical significance. Our meta-analysis also evidenced that Perfectionistic Concerns are linked to more severe binge eating symptomatology than Perfectionistic Strivings, whose association with binge eating appears to be negligible. Accordingly, our findings underscore the importance of addressing perfectionism, particularly Perfectionistic Concerns, as a potential risk factor for binge eating symptomatology.

### Supplementary Information

**Additional file 1:** Dataset.

## Data Availability

The data that supports the findings of this study are available in the supplementary material of this article.

## Appendix 1 Characteristics of studies included in the meta-analysis

**Table:**

Study	Sample							Measures	
	*N*	Sample type	Female %	*M*_age_	*SD*_age_	Nationality	Design	Perfectionism	Binge Eating
Bardone-Cone et al. [4]	406	Community: undergraduates	100	18.7	0.97	USA	Longitudinal	EDI: Perfectionism	EDE-Q: *N* of weeks with BE
Bardone-Cone et al. [5] *Sample 1*	180	Clinical: bulimic with binge eating	100	25.67	8.85	USA	Longitudinal	FMPS: Concern Over Mistakes FMPS: Personal Standards	EDE-Q: Frequency of BE
Bardone-Cone et al. [5] *Sample 2*	169	Clinical: bulimic with vomiting	100	25.67	8.85	USA	Longitudinal	FMPS: Concern Over Mistakes FMPS: Personal Standards	EDE-Q- Frequency of BE
Bardone-Cone et al. [7]	426	Community: undergraduates	100	18.6	0.97	USA	Longitudinal	HMPS: Socially Prescribed Perfectionism HMPS: Self-Oriented Perfectionism	ESS-Negative Affect (an item subset)
Boone et al. [11]	100	Community: undergraduates	100	20.6	2.24	Belgium	Cross-sectional	FMPS: Concern Over Mistakes + Doubts About Actions FMPS: Personal Standards	EDI-2: Bulimia (4 items about BE)
Boone [10]	326	Community: high school students	57	17.1	1.13	Belgium	Cross-sectional	PSPS: Perfectionistic self-promotion HMPS: Socially Prescribed Perfectionism	EDI-2: Bulimia (6 items about BE)
Boone et al. [12]	566	Community: high school students	71	13.28	0.89	Belgium	Cross-sectional and Longitudinal	FMPS: Concern Over Mistakes + Doubts About Actions	EDI-2: Bulimia (6 items about BE)
Brosof and Levinson [15]	300	Community: undergraduates	100	18	1.05	USA	Cross-sectional and Longitudinal	FMPS: Concern Over Mistakes	EDI-2: Bulimia (5 items about BE)
Eddy et al. [24]	122	Clinical: overweight or risk	55,74	11.49	2.3	USA	Cross-sectional	EDI-2: Perfectionism	BMS: BE
Forney et al. [36]	89	Community: undergraduates	93.26	20.38	1.31	USA	Cross-sectional	M-CUP: Reactivity to Mistakes	EPSI: BE
Groleau et al. [46]	176	Clinical: bulimia	100	24.95	5.52	Canada	Cross-sectional	EDI-2: Perfectionism	EDE: Frequency of BE
Jones and Crowther [55]	237	Community: undergraduates	100	19.4	3.9	USA	Cross-sectional	EDI: Perfectionism	BE = 1 No BE = 0
Lampard et al. [59]	162	Clinical: bulimia	100	26.2	7.6	Australia	Cross-sectional	EDI-2: Self-Oriented Perfectionism	EDE: Frequency of BE
Lou et al. [64]	407	Community: adults	47	38.24	13.51	USA	Cross-sectional	FMPS: Concern Over Mistakes FMPS: Personal Standards FMPS: Parental Criticism	Frequency of BE
Mackinnon et al. [65]	200	Community: undergraduates	100	19.86	3.02	USA	Cross-sectional and Longitudinal	SF-FMPS: Concern Over Mistakes SF-FMPS: Personal Standards	EDDS: BE (7 items)
Merwin et al. [67]	263	Community: emerging adults	79,80	21.37	1.89	Canada	Longitudinal	SF-HMPS: Self-Oriented Perfectionism SF-HMPS: Socially Prescribed Perfectionism SF-HMPS: Other Oriented Perfectionism	EDDS: BE (7 items)
Migliore [68]	126	Clinical: binge eaters	100	–	–	USA	Cross-sectional	HMPS: Self-Oriented Perfectionism HMPS: Socially Prescribed Perfectionism	EDE-Q 6.0: BE
Minnich et al. [69]	302	Community: undergraduates	0	19.2	1.3	USA	Cross-sectional and Longitudinal	EDI: Perfectionism	BES
Mushquash y Sherry [70]	317	Community: undergraduates	77,92	20.32	4.34	Canada	Cross-sectional	EDI: Perfectionism APS-R: Perfectionistic Discrepancies DEQ-R: Perfectionistic Discrepancies MDI: Perfectionistic Discrepancies FMPS: Parental Expectation FMPS: Parental Criticism PSPS HMPS: Socially Prescribed Perfectionism HMPS: Self-Oriented Perfectionism	EDI: Bulimia (4 items about BE)
Mushquash y Sherry [71]	218	Community: undergraduates	100	19.99	3.15	Canada	Cross-sectional and Longitudinal	SF-HMPS: Socially Prescribed Perfectionism (items referred to mothers)	EDI: Bulimia (an item subset focused on BE)
Pratt et al. [78]	219	Clinical: obese with binge eating disorder	100	–	–	USA	Cross-sectional	HMPS: Self-Oriented Perfectionism HMPS: Socially Prescribed Perfectionism HMPS: Other Oriented Perfectionism	BES
Rosenberger et al. [80]	131	Clinical: severely obese	100	41.8	10.9	USA	Cross-sectional	EDI: Perfectionism	EDE-Q: Frequency of BE
Sehm y Warschburger [84]	516	Community: adolescents	0	14.37	1.56	Germany	Cross-sectional and Longitudinal	EDI-C: Perfectionism	ChEDE-Q: *N* of days with BE
Sherry et al. [88]	524	Community: undergraduates	81,11	20.09	3.43	Canada and UK	Cross-sectional	FMPS: Concern Over Mistakes FMPS: Doubts About Actions DEQ: Self-Criticism HMPS: Socially Prescribed Perfectionism	EDI: Bulimia (4 items about BE)
Sherry and Hall [85]	566	Community: undergraduates	100	–	–	Canada	Cross-sectional	HMPS: Self-Oriented Perfectionist HMPS + FMPS + EDI: Socially Prescribed Perfectionism MDI: Interpersonal Discrepancies	BULIT-R + EDI + EDDS: a subset of BE items
Short et al. [89]	317	Community: undergraduates	77,92	20.32	4.34	Canada	Cross-sectional	FMPS: Doubts About Actions	EDDS: BE
Slane et al. [90] *Sample 1*	344	Community: twins	100	20.89	2.33	USA	Cross-sectional	FMPS: Concern Over Mistakes	MEBS: BE
Slane et al. [90] *Sample 2*	216	Community: twins	0	20.89	2.33	USA	Cross-sectional	FMPS: Concern Over Mistakes	MEBS: BE
Smith et al. [92]	200	Community: undergraduates	100	19.9	3.02	Canada	Cross-sectional and Longitudinal	SF-FMPS: Concern Over Mistakes SF-FMPS: Doubts About Actions SF-HMPS: Socially Prescribed Perfectionism	BULIT-R + EDI + EDDS: a subset of BE items
Watson et al. [105]* Sample 1*	238	Clinical: eating disorder with binge eating	100	26.03	8.5	Canada	Longitudinal	EDI: Self-Oriented Perfectionism + FMPS: Personal Standards	EDE-Q: Frequency of BE
Watson et al. [105]* Sample 2*	258	Clinical: eating disorder with purging	100	26.03	8.5	Canada	Longitudinal	EDI: Self-Oriented Perfectionism + FMPS: Personal Standards	EDE-Q: Frequency of BE
Welch et al. [106]	520	Community: undergraduates	100	20.89	4.43	Canada	Cross-sectional	EDI-2: Perfectionism HMPS: Self-Oriented Perfectionism HMPS: Socially Prescribed Perfectionism HMPS: Other Oriented Perfectionism	Binge Scale
Zelkowitz and Cole [108]	251	Community: undergraduates	79,50	19.1	1.23	USA	Cross-sectional	DEQ: Self-Criticism + SRS + FSC: Self-Criticism	EDE-Q: Frequency of BE

EDI, Eating Disorder Inventory [41], EDE-Q, Eating Disorder Examination Questionnaire [25], ESS, Eating Self-Efficacy Scale [43], EDI-2, Eating Disorder Inventory [40], BMS, Bulimic Modeling Scale [96], M-CUP, Measure of Constructs Underlying Perfectionism [94], EPSI, Eating Pathology Symptom Inventory [35], EDE, Eating Disorders Examination [27], EDDS, Eating Disorder Diagnostic Scale [97], EDE-Q 6.0, Eating Disorder Examination Questionnaire 6.0 [26], BES, Binge Eating Scale [45], ChEDE-Q, Child Eating Disorder Examination-Questionnaire [53], BULIT-R, Bulimia Test-Revised [63], MEBS, Minnesota Eating Behavior Survey [79], Binge Scale [47], FSC, Self-Criticism/Self-Reassurance Scale [42], SRS, Self-Rating Scale [54], FMPS, Frost’s Multidimensional Perfectionism Scale [39], SF-FMPS, The Short Form of the Frost’s Multidimensional Perfectionism Scale [21], PSPS, Perfectionistic Self-Presentation Scale [51], HMPS, Hewitt's Multidimensional Perfectionism Scale [49], SF-HMPS, The Short Form of the Hewitt’s Multidimensional Perfectionism Scale [52], EDI-C, Eating Disorder Inventory-Children [102], DEQ, Depressive Experiencies Questionnaire [8], DEQ-R, Reconstructed Depressive Experiences Questionnaire [3], APS-R, Almost Perfect Scale-Revised [91], MDI, Multidimensional Discrepancy Inventory (MDI, Flett and Hewitt [31], BE, Binge Eating.
